# Updated rapid risk assessment from ECDC on coronavirus disease 2019 (COVID-19) pandemic: increased transmission in the EU/EEA and the UK

**DOI:** 10.2807/1560-7917.ES.2020.25.12.2003261

**Published:** 2020-03-26

**Authors:** Eurosurveillance editorial team

**Affiliations:** 1 European Centre for Disease Prevention and Control (ECDC), Stockholm, Sweden

The European Centre for Disease Prevention and Control (ECDC) provides regularly updated information on coronavirus disease-2019 (COVID-19) relevant to Europe on a dedicated webpage. Besides general information including Q&As, daily case counts, and maps with disease distribution, examples of latest updates comprise: Considerations related to the safe handling of bodies of deceased persons with suspected or confirmed COVID-19 and Coronavirus disease 2019 (COVID-19) and supply of substances of human origin in the EU/EEA. ECDC also publishes regular risk assessments and the Box below contains the summary from the seventh update published on 25 March 2020.

Box **Summary of the ECDC rapid risk assessment from 25 March 2020**

On 31 December 2019, a cluster of pneumonia cases of unknown aetiology was reported in Wuhan, Hubei Province, China. On 9 January 2020, China CDC reported a novel coronavirus as the causative agent of this outbreak, coronavirus disease 2019 (COVID-19).

As of 25 March 2020, more than 416 916 cases of COVID-19 were reported worldwide by more than 150 countries. An increasing proportion of global cases are from EU/EEA countries and the UK. As of 25 March, 204 930 cases and 11 810 deaths have been reported in the EU/EEA and the UK. The number of reported COVID-19 cases is rapidly increasing in all EU/EEA countries and the UK, and the notification rate is increasing at similar trajectory as was observed in Hubei province in late January/early February and in Italy in late February/early March.

Clinical presentations of COVID-19 range from no symptoms (asymptomatic) to severe pneumonia; severe disease can lead to death. In EU/EEA countries with available data, 30% of diagnosed COVID-19 cases were hospitalised and 4% had severe illness. Hospitalisation rates were higher for those aged 60 years and above. Estimates of crude case-fatality for Germany, Italy and Spain showed that both the risk and absolute numbers of deaths rapidly increased with age for those aged 60 years and above in each country. Among hospitalised cases, severe illness was reported in 15% of cases, and death occurred in 12% of these cases, with higher case–fatality rates in older adults.

In the present situation where COVID-19 is rapidly spreading in Europe, the current assessment is:

- The risk of severe disease associated with COVID-19 for people in the EU/EEA and the UK is currently considered moderate for the general population and very high for older adults and individuals with chronic underlying conditions.
- The risk of occurrence of widespread national community transmission of COVID-19 in the EU/EEA and the UK in the coming weeks is moderate if effective mitigation measures are in place and very high if insufficient mitigation measures are in place.
- The risk of healthcare system capacity being exceeded in the EU/EEA and the UK in the coming weeks is considered high.

Measures taken at this stage should ultimately aim at protecting the most vulnerable population groups from severe illness and fatal outcome by reducing transmission in the general population and enabling the reinforcement of healthcare systems. Given the current epidemiology and risk assessment, and the expected developments in the next days to few weeks, the following public health measures to reduce further spread and mitigate the impact of the pandemic should be applied in EU/EEA countries:

**Community measures and social distancing** should be implemented proactively and with active community engagement in order to reduce the impact of the epidemic and to delay its peak, allowing healthcare systems to prepare and cope with an increased influx of patients.

- Rigorous hand washing, respiratory etiquette, and the use of face masks by persons with respiratory symptoms can contribute to decreasing the spread of COVID-19 in the community.
- Layered application of social distancing measures (including isolation of cases and quarantine of contacts; measures at, or closure of, workplaces and educational institutions; restrictions in movement and social gatherings) can play a significant role in reducing community transmission if strictly adhered to.

**Measures in healthcare facilities** are an immediate priority in order to: 1) slow the demand for specialised healthcare, such as ICU beds; 2) safeguard risk groups 3); protect healthcare workers that provide care; and 4) minimise the export of cases to other healthcare facilities and the community.

- In healthcare settings, surge capacity plans must be available and up-to-date in expectation for the high demand for care of patients with moderate or severe respiratory distress. Critical care needs can be required for up to 15% of hospitalised patients with COVID-19.
- Long-term care facilities should implement infection prevention and control measures.
- Healthcare workers need to be protected as they are part of the critical infrastructure of response to this epidemic and should be prioritised in the testing policy; healthcare workers need access to, and appropriate training on, PPE use.
- Cohorting of hospitalised cases is advised to save staff and PPE resources.
- Rational use of PPE should be employed at all times, but especially when there is shortage of PPE material.
- Patients with mild clinical presentation, particularly those who are not in a recognised risk group for developing severe disease, can be managed at home with instructions to follow up if symptoms deteriorate. Measures to prevent household transmission should be advised and/or facilitated.
- Patients presenting with respiratory distress with increased need for oxygenation require management in hospital. Patients in critical condition need specialised care, on average for more than two weeks.
- Current criteria for discharge from the hospital include resolution of symptoms and laboratory evidence of SARS-CoV-2 clearance from the upper respiratory tract. Criteria can be adapted to the local context.

**Testing and surveillance strategies** should rapidly detect cases and elucidate transmission patterns.

- Capacity for SARS-CoV-2 laboratory testing at high levels is essential.
- Shortages in testing capacity need to be anticipated and addressed, taking the needs for testing of other critical diseases into account; if capacity is exceeded, priority should be given to the testing of vulnerable patients, healthcare workers and patients requiring hospitalisation.
- Validation of performance and operational utility of selected rapid/point-of-care tests (e.g. for antigen detection) is needed before recommending their use for clinical diagnosis.
- Serological assays are currently not recommended for case detection.
- Sentinel syndromic and virological surveillance of ARI/ILI allows for the monitoring of community transmission and, together with surveillance of hospitalised cases, can help to define triggers for escalation/de-escalation of mitigation measures.
- Countries recommending that patients with ARI/ILI should not visit general practitioners need to identify alternative sources for community-based surveillance such as telephone helplines.
- Hospital-based surveillance is needed to identify risk groups for severe disease, measure impact and inform decisions on mitigation measures.
- Contact tracing should continue during all stages of the epidemic as long as resources allow. For areas with widespread transmission there is still value in continuing contact tracing, resources permitting, as part of a range of measures.

A strategic approach based on early and rigorous application of these measures will help reduce the burden and pressure on the healthcare system, and in particular on hospitals, and will allow more time for the testing of therapeutics and vaccine development.

What is new in this update?

- Updated data on the epidemiological situation in the EU/EEA and the UK
- Data on disease and case severity from Europe
- Risk associated with COVID-19 for people from the EU/EEA and the UK
- Risk of widespread national community transmission in the EU/EEA and the UK in the coming weeks
- Risk to healthcare systems capacity being exceeded in the EU/EEA and the UK in the coming weeks
- Options for preparedness and response for the mitigation phase focused on the community setting, hospitals, and surveillance and testing.

Source: European Centre for Disease Prevention and Control (ECDC). Rapid risk assessment: Novel coronavirus disease 2019 (COVID-19) pandemic: increased transmission in the EU/EEA and the UK – seventh update. ECDC: Stockholm; 25 March 2020. Available from: https://www.ecdc.europa.eu/sites/default/files/documents/RRA-seventh-update-Outbreak-of-coronavirus-disease-COVID-19.pdf
ARI/ILI: acute respiratory infection/ influenza-like-illness; COVID: coronavirus disease; ECDC: European Centre for Disease Prevention and Control; EU/EEA: European Union/European Economic Area; SARS-CoV: severe acute respiratory syndrome coronavirus; UK: United Kingdom.

